# Nuclear mechanosignaling in striated muscle diseases

**DOI:** 10.3389/fphys.2023.1126111

**Published:** 2023-03-07

**Authors:** Bo Zhang, Joseph D. Powers, Andrew D. McCulloch, Neil C. Chi

**Affiliations:** ^1^ Department of Bioengineering, University of California San Diego, La Jolla, CA, United States; ^2^ Institute for Engineering in Medicine, University of California San Diego, La Jolla, CA, United States; ^3^ Department of Medicine, Division of Cardiovascular Medicine, University of California San Diego, La Jolla, CA, United States; ^4^ Institute of Genomic Medicine, University of California San Diego, La Jolla, CA, United States

**Keywords:** mechanosignaling, nucleus, myocytes, nucleoskeleton, nuclear morphology, LINC complex, cardiomyopathy, laminopathy

## Abstract

Mechanosignaling describes processes by which biomechanical stimuli are transduced into cellular responses. External biophysical forces can be transmitted *via* structural protein networks that span from the cellular membrane to the cytoskeleton and the nucleus, where they can regulate gene expression through a series of biomechanical and/or biochemical mechanosensitive mechanisms, including chromatin remodeling, translocation of transcriptional regulators, and epigenetic factors. Striated muscle cells, including cardiac and skeletal muscle myocytes, utilize these nuclear mechanosignaling mechanisms to respond to changes in their intracellular and extracellular mechanical environment and mediate gene expression and cell remodeling. In this brief review, we highlight and discuss recent experimental work focused on the pathway of biomechanical stimulus propagation at the nucleus-cytoskeleton interface of striated muscles, and the mechanisms by which these pathways regulate gene regulation, muscle structure, and function. Furthermore, we discuss nuclear protein mutations that affect mechanosignaling function in human and animal models of cardiomyopathy. Furthermore, current open questions and future challenges in investigating striated muscle nuclear mechanosignaling are further discussed.

## 1 Introduction

Striated muscles, including cardiac muscle, skeletal muscle, or insect flight muscles, are highly organized tissues specifically designed to produce precise forces and movements for a diverse set of functions. Striated muscle cells (myocytes) comprising these tissues all share the remarkable ability to both produce and adapt to highly variable microenvironmental forces. During myocyte contraction, ATP-driven myosin motors on the thick filament of the sarcomere, the contractile unit of myocytes, cyclically interact with actin filaments to induce filament sliding and force-generating sarcomere shortening (Powers et al., 2021). For these molecular-level processes to translate to cell- and tissue-level force production, sarcomere-generated forces must be transmitted from the sarcomere, to the intermediate filaments of the cytoskeleton and costamere, and outward to the extracellular matrix (ECM) and surrounding cells. Similarly, forces generated from outside the cell (e.g., passive stretch, ECM stiffness) are transmitted into and distributed throughout the cell, putatively through the same molecular structures.

Both intracellular and extracellular forces ultimately reach the myocyte nucleus, where they can be processed into biological responses that mediate the structural or functional adaptation of the cell through a process called nuclear mechanosignaling. The nucleus of striated muscle cells, among many other cell types (Maurer and Lammerding, 2019), is now considered to be a key mechanosensor, reacting to forces at the nucleus-cytoskeleton interface and mediating downstream biological responses. Factors such as nuclear envelope structure, intracellular nuclear positioning, and nuclear deformation can influence the mechanosensing ability and response to mechanical forces (Jabre et al., 2021). Therefore, myocytes and their nuclei must maintain a mechanical homeostasis during muscle contractions and deformations to preserve healthy muscle structure and function.

However, this homeostasis can be disrupted by defects in the ability of the myocyte nucleus to sense the mechanical forces and/or by abnormal mechanical forces imposed onto the nucleus. Myocytes under persistent abnormal mechanical forces, such as chronically altered contractility or abnormal strains imposed during passive muscle stretch, can trigger a myriad of biological responses that often lead to maladaptive cell remodeling or hypertrophy, contractile dysfunction, and cell death (Dupont et al., 2011; Kresh and Chopra, 2011). In mammalian hearts, for example, prolonged high load or deposition of extracellular matrix (ECM) can lead to cardiac hypertrophy, cardiomyopathy, and heart failure (Cook et al., 2014; Lyon et al., 2015; Yadav et al., 2021). Moreover, skeletal muscle cells with defective proteins that mediate the structural integrity of the nucleoskeleton or the ability of the nucleus to sense mechanical loading can lead to muscular dystrophy phenotypes (Bianchi et al., 2018).

Elucidating the role of nuclear mechanosignaling in mechanosensitive genetic signaling pathways and their involvement in muscular diseases is currently a major research area, but there are still many unknown mechanisms about the transduction of intracellular and extracellular forces into downstream gene expression *via* nuclear mechanosignaling. Here, we briefly review our current understanding of mechanisms by which mechanical forces and nuclear mechanosensing influence gene regulatory programs underlying pathological remodeling and dysfunction of striated muscle cells.

## 2 The bridge between chromatin and the extracellular matrix of striated muscle cells

**FIGURE 1:**
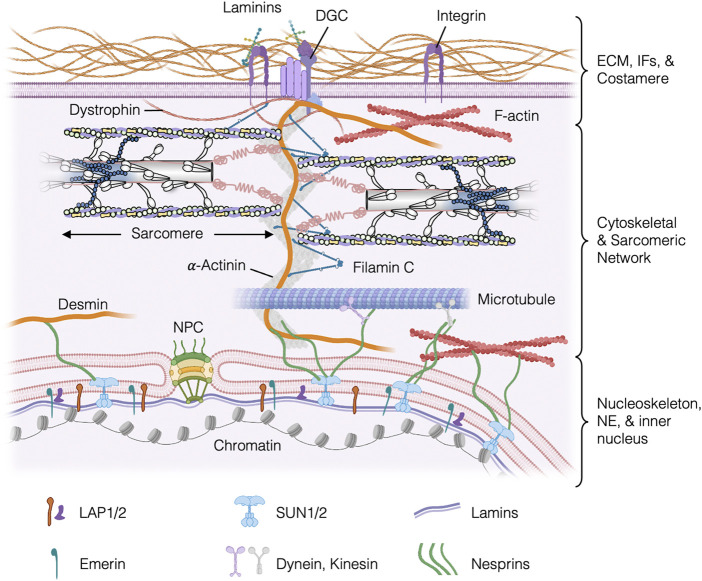
Schematic of the intracellular environment of a mature cardiomyocyte, illustrating mechanical coupling between the ECM the costamere, the sarcomere, the nucleoskeleton, and chromatin. Costameres consists of integrins, laminins, and DGCs (among other proteins not depicted here) and provides linkages between the Z-disks of the sarcomere, IFs in the cortical cytoskeleton, and the ECM, and other cytoskeletal IFs. Desmin, a-actinin, and Filamin C provide (among other proteins not shown here for clarity) provide structural integrity to the Z-disk, to which myofilaments of the sarcomere are anchored. ECM, extracellular matrix; IFs, intermediate filaments; DGC, dystrophin-glycoprotein complex; LAP1/2, lamina-associated polypeptide 1/2; NPC, nuclear pore complex; NE, nuclear envelope.

In healthy striated muscle cells, the pseudo-crystalline structure of myofilaments, sarcomeres, and myofibrils establishes a highly organized and uniquely anisotropic subcellular architecture. This subcellular organization is, in part, maintained by rapid and spatially controlled protein turnover (Wood et al., 2022) of large macromolecular complexes, which together provide mechanical linkages between chromatin, the cytoskeletal and sarcomere network, and the extracellular matrix (Figure 1). At the cell membrane-ECM interface, the cell connection to the ECM is supported by transmembrane protein complexes called costameres. As depicted in Figure 1, the costamere is a molecular complex that consists of integrin, vinculin, integrin-linked kinase, ankyrin, filamin C, talin, desmin, dystrophin, the dystrophin-glycoprotein complex (DGC), among many others (Bang et al., 2022). The costamere also helps connect the Z-disk of the sarcomere to the sarcolemma and the ECM, providing a structural and mechanical link between the intracellular contractile machinery and the extracellular environment. Deeper into the cell interior, a framework of microtubules, intermediate filaments, and F-actin maintain the connection of the Z-disks throughout the cytoskeleton and to the nucleoskeleton.

The correct crosstalk between cytoskeleton and nucleoskeleton is necessary for proper mechanosignaling in straited muscle cells. Muscle-specific LIM domain protein (MLP) has been shown to be a stretch sensor in cardiac muscle in both mice and human (Knöll et al., 2002). MLP KO mice showed severe DCM phenotype and mRNA profile. Cardiomyocytes from these mice indicate significant disruption in their cardiomyocyte cytoarchitecture and myofibril arrays (Arber et al., 1997). It is thought that MLP-TCAP binding is necessary for proper cardiac muscle mechanotransduction. Loss of MLP-TCAP changes intrinsic elastic properties of titin, which further affects proper mechanotransduction from the ECM to the nucleus (Arber et al., 1997; Knöll et al., 2002). Another example of cytoskeleton-nucleoskeleton crosstalk is desmin [please see (Capetanaki et al., 2015; Hnia et al., 2015; Tsikitis et al., 2018) for more comprehensive reviews on desmin in muscle diseases]. Desmin interfilament cytoskeleton network connects with the lamin nucleoskeleton either directly through the nuclear pores or indirectly through plectin-nesprin3-LINC complex (Georgatos et al., 1987; Lockard and Bloom, 1993; Frock et al., 2006). This crosstalk is essential for propagating mechanical stimuli from ECM to the nucleus. Depletion of either desmin or Nesprin-3 causes nuclear envelope deformation and chromatin disorganization (also discussed in Section 4, below). Moreover, desmin-KD cardiomyocytes showed an altered expression profile that has significant cardiac dysfunction, DCM, hypertrophy signatures (Heffler et al., 2020). Patients with dysfunctional desmin mutations showed granulofilamentous accumulations and sandwich formations and characteristic z-disk deformity (Vrabie et al., 2005; Claeys et al., 2008). These abnormal structures might affect cytoskeleton and nucleoskeleton crosstalk. Furthermore, in addition to nuclear mechanical regulation, desmin also serves as a signaling platform to help its binding partners in significant cellular functions like DNA repair (with MLH1), endocytosis pathway (with ITSN1), and calcium hemostasis (with S100A1) (Hnia et al., 2015).

The primary connection between the nucleus and cytoskeleton in striated muscles is a protein complex called the Linker of Nucleoskeleton and Cytoskeleton (LINC) complex, which comprise many proteins that have been associated with cardiomyopathies, demonstrating its critical role in maintaining striated muscle function [please see (Stroud et al., 2014; Stroud, 2018; Bang et al., 2022) for reviews on this subject]. As illustrated in Figure 1, the LINC complex is part of the molecular connections between the nuclear lamina and the cytoskeletal/sarcomeric network, which is facilitated (in part) by nesprins at the outer nuclear membrane and SUN domain proteins (SUN1/2) at the inner nuclear membrane. In the inner nucleus, chromatin interacts directly with the LINC complex *via* emerin, lamins, and many other proteins. In particular, the interaction between lamin and desmin in cardiomyocytes is critical for normal heart function. A lack of lamin A/C expression in mice cardiomyocytes has been shown to exhibit disorganized desmin filaments and paranuclear regions where the desmin filaments are detached from the nucleus (Nikolova et al., 2004). Many Lamin A/C mutations also show different degrees of disruption on cytoskeleton structures in both human and mice models (Sebillon et al., 2003; Mounkes et al., 2005; Lanzicher et al., 2015; Yang et al., 2021). In a mouse model of dilated cardiomyopathy caused by the lamin A/C mutation H222P, desmin loses its localization from Z-disks and intercalated disks and forms aggregates (Galata et al., 2018). Interestingly, ameliorating the desmin-associated defects in these cardiomyocytes provided cardioprotection from the H222P lamin A/C mutation (Galata et al., 2018), suggesting that desmin is a promising target for certain laminopathies.

Thus, myocytes, like many other cell types, exhibit tight mechanical coupling from chromatin to ECM. However, the anisotropic subcellular ultrastructure of myocytes and their high degree of force production impose distinct mechanical environments onto myonuclei that may elicit unique and underexplored nuclear mechanosignaling pathways underlying muscle structure and function. In the sections that follow, we discuss specific proteins known to be involved in the maintenance of this process, and the relationship between defects in nuclear mechanosignaling, mechanosensitive gene regulation and protein synthesis, and striated muscle diseases.

## 3 Functions of nuclear envelope and nucleoskeleton proteins in healthy and diseased myocytes

The nuclear envelope (NE) and nucleoskeleton provide a connection between the sarcomere, the cytoskeleton, and chromatin in myocytes, which likely facilitates important mechanosensitive gene regulatory programs. In the following subsections, we discuss specific proteins known to be involved in nuclear mechanosignaling or nucleus-related pathologies in striated muscle.

### 3.1 Nesprins

Nesprins provide many of the linkages between the outer nuclear membrane and cytoskeletal proteins. Nesprins generally are composed of three domains: a highly conserved C-terminus which interacts with the SUN (Sad1p, UNC-84) protein at the outer nuclear membrane (ONM), a spectrin repeat domain, and a diverse N-terminus that binds to cytoskeleton structures (Stroud, 2018; Liao et al., 2019). Nesprin-1/2 can also directly bind to actin filaments (Chancellor et al., 2010; Zhou et al., 2018), while nesprin-1α2 indirectly interacts with microtubule structures through Kinesin 1 (Mislow et al., 2002; Briand and Collas, 2020). Nesprin-3 indirectly binds to intermediate filaments through plectin (Wilhelmsen et al., 2005), and microtubules through Microtubule-Actin Cross-linking Factor (MACF) and Bullous pemphigoid antigen 1 (BPAG1) (Ketema and Sonnenberg, 2011; Liao et al., 2019). The interaction between Nesprin and SUN forms the basic structure of LINC that can propagate forces between the cytoskeleton and the nucleus.

Nesprin-1/2 mutations have been found to be correlated with Emery-Dreifuss muscular dystrophy (EDMD) and dilated cardiomyopathy (DCM) (Zhang et al., 2007; Puckelwartz et al., 2010). One patient with mutation in the C-terminus of nesprin-1 (p.R374H) experiences DCM with elevated expression of nesprin-1 and lamin A/C (Puckelwartz et al., 2010). Mice that have C-terminus KASH-domain deleted in nesprin-1 developed EDMD-like and DCM phenotypes. Gene-edited mice where the C-terminus of nesprin-1 was deleted experience hindlimb muscle weakness and cardiac conduction defects (Puckelwartz et al., 2009). Nesprin-1/2 siRNA knockdown in fibroblasts leads to abnormal nuclear morphology and mislocalization of emerin and SUN-2 (Zhang et al., 2007). Surprisingly, nesprin-3 mutation, deletion, or overexpression in mice and zebrafish does not seem to cause significant phenotypes (Banerjee et al., 2014).

### 3.2 SUN proteins

SUN1/2 are expressed in skeletal and cardiac muscles in humans (Crisp et al., 2006; Puckelwartz et al., 2009; Zhang et al., 2010). They both have a C-terminal SUN domain, a coiled-coil domain, a transmembrane (TM) domain, and an N-terminus nucleoplasmic domain (Stroud, 2018; Sosa Ponce et al., 2020). The SUN domain is the most conserved domain that interacts with the KASH domain on Nesprin (Sosa et al., 2012; Wang et al., 2012; Sosa Ponce et al., 2020). Inter-protein interactions between the coiled-coil domains form the SUN protein trimer (Sosa et al., 2012; Jahed et al., 2018a; Jahed et al., 2018b; Sosa Ponce et al., 2020). The N-terminus nucleoplasm domain binds with emerin and lamin A/C, anchoring the SUN protein at the NE (Crisp et al., 2006; Haque et al., 2010).

SUN1/2 mutations are rarely associated with cardiomyopathy (Meinke et al., 2014; Bang et al., 2022). However, patients carrying SUN mutations can experience significant cardiac abnormalities when there are also mutations in other LINC proteins (Manila et al., 1998; Bonne et al., 2000; Vytopil et al., 2003). Neither global knockout (KO) of SUN1/2 in mice show significant defects in heart or skeletal muscle (Ding et al., 2007; Lei et al., 2009). However, double-KO of SUN1/2 in mice leads to premature death due to central neuron system defects (Lei et al., 2009). Nesprin-1 but not lamin A/C are mislocalized in SUN1/2 double-KO mice skeletal muscle cells (Lei et al., 2009). These results indicate that SUN1 and SUN2 play a critical but potentially redundant role in Nesprin-1 localization on skeletal muscle NE.

### 3.3 Emerin

Emerin has a long N-terminus in the nucleoplasmic domain that interacts with SUN1/2, LAP1, lamin A/C, histone deacetylase 3 (HDAC3), and barrier to autointegration factor (BAF) (Lee et al., 2001; Demmerle et al., 2012; Shin et al., 2013; Berk et al., 2014). HDAC3 and BAF help emerin indirectly bind with chromatin, and the lamin A/C and LAP1 interaction with emerin is thought to be important for its localization at inner nuclear membrane (INM) (Mislow et al., 2002; Haque et al., 2010; Guilluy et al., 2014; Shin et al., 2014; Osmanagic-Myers et al., 2015).

Mutations in emerin are clinically observed to be related to EDMD, DCM, and other cardiac and skeletal muscle defects (Bione et al., 1994; Bione et al., 1995; Essawy et al., 2019). Emerin KO mice were recently developed to study the effect of loss of emerin (Lammerding et al., 2005; Melcon et al., 2006). Surprisingly, emerin KO mice show no significant difference in skeletal muscle development and only a mild reduction in ejection fraction (Melcon et al., 2006; Ozawa et al., 2006; Stubenvoll et al., 2015). However, LAP1 and emerin double-KO mice showed exacerbated myopathy, suggesting that LAP1 and emerin may have compensatory functions in myocytes (Shin et al., 2013).

### 3.4 Lamina-associated polypeptides (LAP proteins)

Lamina-associated polypeptide 1 (LAP1) is encoded by the gene *TOR1AIP1*. In humans, two isoforms of LAP1 (LAP1B and LAP1C) are expressed through alternative splicing (Kondo et al., 2002; Santos et al., 2014). Both LAP1 isoforms locates at the INM and LAP1B has an N-terminus nucleoplasmic domain that interacts with Emerin, lamin A/C, lamin B1, and chromatin (Foisner and Gerace, 1993; Shin et al., 2013; Luithle et al., 2020). It also has a C-terminus that interacts with Torsin A, B, 2, and 3 (Goodchild and Dauer, 2005; Jungwirth et al., 2010; Kim et al., 2010). Because of its close interaction with mechanosignaling proteins at the INM, LAP1 is suspected to be involved in the nuclear mechanosensing (Shin et al., 2013). However, the exact mechanism of LAP1 specific mechanosignaling remains unknown.

Patients with LAP1 mutation p.Arg321* showed significant decrease in expression of both LAP1 isoforms in mRNA and Protein level (Fichtman et al., 2019). This specific mutation truncates the entire transmembrane domain and cytoplasmic domain. Skin fibroblast from patients showed distorted nuclear shape and abnormal lamin A/C localizations. Patients also suffer from congenital heart malformations and a series of other abnormalities. Another frameshift LAP1 mutation (c.186delG) leads to an early stop codon (Kayman-Kurekci et al., 2014). The consanguineous patients carrying this mutation showed muscle weakness and atrophy, rigid spine, and rigid interphalangeal hand joints. Only one patient showed decreased cardiac function. Interestingly, this mutation only exists at the LAP1B isoform while LAP1C is unaffected. Interestingly, patients’ skeletal muscle biopsy showed significantly reduced LAP1B and slightly increased LAP1C expression. The biopsy also showed intact sarcomere organization but fragmented nuclear membrane, nuclear blebbing, and naked chromatin. Because of the relatively mild phenotypes in this isoform specific mutation, this study suggests that LAP1C might be complementary to LAP1B function and LAP1B is important for nuclear structure maintenance (Kayman-Kurekci et al., 2014). Furthermore, mice with conditional LAP1 KO in striated muscle cells experienced significant cardiac and skeletal myopathy (Shin et al., 2013). Skin fibroblasts from global LAP1-KO mice showed co-mislocalization of emerin and lamin A/C, indicating the potential role of LAP1 in localizing LINC proteins.

LAP2 has six isoforms derived from alternative splicing. All of the six isoforms have an N-terminus with a LEM (LAP2, emerin, MAN1) domain that interacts with BAF that further binds with chromatin (Lin et al., 2000). The C-terminus of each LAP2 protein is highly variable in length and possesses different functions. For example, LAP2α has the longest C-terminus domain which lacks a TM domain but interacts with lamin A/C, while LAP2β interacts with lamin B1 at the INM and possesses a TM domain (Foisner and Gerace, 1993; Dechat et al., 1998). For more details about isoform domains and protein interactions, we recommend the following review (Dechat et al., 2000a).

Patients with LAP2α p.R690C mutation have been reported with severe DCM (Taylor et al., 2005). This mutation occurs within the domain where LAP2α interacts with lamin A/C, and *in vitro* functional analysis shows interaction of mutated LAP2α with the pre-lamin A is significantly compromised compared to the wild-type LAP2α. Interaction of lamin A/C and LAP2α seems to be the common mechanism for LAP2α mutations that lead to diseases in other systems as well (Pekovic et al., 2007; Gesson et al., 2014; Vidak et al., 2018).

### 3.5 Nuclear pore complex (NPC)

The nuclear pore complex (NPC) mediates the transport of all macromolecules between the nucleus and the sarcoplasm. NPC is composed of ∼30 different proteins with multiple copies of each nucleoporin (Cronshaw et al., 2002), and these nucleoporin constitute three different regions: the cytoplasmic ring, the central framework, and the nuclear basket (Hurt and Beck, 2015). NUP153 is a nucleoporin on the nuclear basket that interacts with SUN1, lamin A/C and lamin B (Liu et al., 2007; Al-Haboubi et al., 2011; Li and Noegel, 2015). It is suspected that NPCs possess mechano-activation through the NUP153-SUN1 interaction. It is hypothesized that force propagation from SUN1 to NUP153 can induce rearrangement of the basket structure that extends from the NPC into the nucleus (Donnaloja et al., 2019). Depletion of NUP153, which alters the localization of Sun1 and F-actin structure, further supports this hypothesis (Zhou and Panté, 2010).

Mutations on NUP153 are not directly correlated with skeletal or cardiac myopathy according to our knowledge. However, skin fibroblasts with lamin A/C mutations within the Ig-fold domain, which interact with NUP153 shows altered NUP153 localization (LMNA p.453W) (Al-Haboubi et al., 2011). Another mutation on lamin A/C (p.A399C) also disrupts the lamin A/C-NUP155 interaction and causes atrial fibrillation (Han et al., 2019). Interestingly, ischemic and dilated cardiomyopathy hearts harvested from patients showed significant increase in NUP153 (Tarazón et al., 2012), however the mechanism behind NUP153 up-regulation and the downstream effects in these patients is not clear.

## 4 Nuclear morphology, migration, and deformation in striated muscle diseases

It is clear that nuclear morphology and deformation play an important role in regulating gene expression in many different cell types (Thomas et al., 2002; Kalukula et al., 2022). For example, extracellular-based mechanical tension across the nuclear membrane of aortic valve fibroblasts, mediated by the actin cytoskeleton and LINC complex, facilitates chromatin remodeling that leads to pro-fibrotic myofibroblast persistence (Walker et al., 2021). In striated muscle cells, the large magnitude of myocyte deformations experienced during muscle contraction and relaxation may facilitate processes involving nuclear migration, chromatin organization, and mechanosensitive gene expression. For example, during skeletal muscle contractions, centripetal forces exerted by myofibrils facilitate the migration of the nucleus to the periphery of the myofibers (Roman et al., 2017). Furthermore, myonuclei are readily localized *via* cytoskeletal protein networks to sites of myocyte damage to accelerate sarcomere repair (Roman et al., 2021). Moreover, physiological myonuclear deformations occur during normal contractions and passive stretches (Kalukula et al., 2022). A recent study (Ghosh et al., 2019) demonstrated that intranuclear deformations during cardiomyocyte contraction depends on the mechanical properties of the extracellular environment, and that areas in the nucleus with denser chromatin exhibited greater strain discrepancies between substrate stiffnesses. This supports the hypothesis that myonuclei transduce the mechanical properties of the extracellular environment into epigenetic control *via* intranuclear strain distributions that affect chromatin organization and accessibility.

More compliant and fragile nuclei, as well as decoupled nucleus-cytoskeleton connections, can be consequences of nuclear envelope protein mutations associated with various myopathies (Zwerger et al., 2013). In *Drosophila* larvae with mutated Nesprin, myonuclei deform differently during contractions compared with wild-type flies (Lorber et al., 2020). In an aging context, cardiomyocytes from *Drosophila* also exhibit decreased Lamin C expression with age, which coincides with decreased nuclear size, increased nuclear stiffness, and altered transcriptional programs (Kirkland et al., 2023). Similarly, aged mice and non-human primates exhibit reduced nuclear size and circularity, with old mice also having reduced Lamin C compared to younger mice (Kirkland et al., 2023). Moreover, mutant lamins in skeletal muscle associated with laminopathies and muscular dystrophy cause nuclear envelope rupture and DNA damage that correlate with disease severity in both mice and humans (Earle et al., 2020). However, microtubule stabilization reinforced myonuclei in these diseased muscle fibers, reducing nuclear damage and rescued myocyte function in viability in cells with mutated lamins (Earle et al., 2020).

Defects or mutations in proteins comprising the nucleoskeleton are not only associated with nuclear envelopathies and laminopathies, but they can also lead to altered nuclear morphology and mechanotransduction (Janin and Gache, 2018; Ross and Stroud, 2021). Mice lacking the nuclear envelope transmembrane protein 39 (Net39) develop skeletal muscle dysfunction and remodeling that resembles Emery Dreifuss muscular dystrophy (Ramirez-Martinez et al., 2021). Moreover, the nuclei in skeletal muscle fibers from Net39-null mice exhibit significant deformations, changes in chromatin accessibility, and altered gene expression, while human muscle biopsies from patients with Emery Dreifuss muscular dystrophy revealed a downregulation of Net39 (Ramirez-Martinez et al., 2021). [See (Bianchi et al., 2018) for a recent review of Emery Dreifuss muscular dystrophy in the context of nuclear mechanosignaling.]

In the heart, a loss of desmin or nesprin in the LINC complex in cardiomyocytes causes the nucleus to collapse in response to neighboring polymerizing microtubules, which can lead to chromatin reorganization, DNA damage, and altered transcriptomic expression (Heffler et al., 2020). Similarly, genetically engineered mice carrying the human p.L13 > R mutation in the nuclear envelope protein LEM domain containing protein 2 (LEMD2) develop severe DCM and cardiac fibrosis, which lead to premature death (Caravia et al., 2022). Homozygous mice for the p.L13 > R LEMD2 variant exhibit disorganized heterochromatin, nuclear envelope deformations, extensive DNA damage, and apoptosis linked to p53 activation (Caravia et al., 2022), suggesting that LEMD2 is essential for genome stability and cardiac function.

Finally, we note that defects in cytoskeletal proteins not directly linked to the nuclear envelope (but associated with other cardiomyopathies) also can cause changes in nuclear structure. For example, a loss of filamin C in adult mouse hearts causes nuclear remodeling and altered gene expression (Zhou et al., 2020; Powers et al., 2022; Powers and McCulloch, 2022), suggesting that cytoskeletal proteins not directly interacting with the nucleoskeleton in myocytes may also influence nuclear structure and mechanosignaling.

## 5 Mechanical regulation of chromatin positioning, epigenetic factors, and gene expression in myocytes

In the nucleus, chromatin accessibility and its three-dimensional organization are correlated (Bertero et al., 2019; Bertero and Rosa-Garrido, 2021). As such, mechanical forces and/or defects in the nucleoskeleton structure that affect chromatin organization may be key factors underlying mechanosensitive gene regulation of pathogenic remodeling or dysfunction in myocytes. Lamin has been shown to organize the three-dimensional genome from the nuclear periphery (Zheng et al., 2018). While the conventional view is that chromatin activity is reduced from the nuclear center to periphery, a recent study (Amiad-Pavlov et al., 2021) found that A-type lamin upregulation in instar larva muscle caused chromatin to collapse toward the nuclear center while also reducing overall levels of active chromatin. These findings suggest that: 1) lamina composition regulates the organization of peripheral chromatin, and 2) there may not be a clear spatial distribution of active versus inactive chromatin throughout the nuclear volume. Furthermore, a loss of all isoforms of lamin has been shown to expand or detach specific lamina-associated domains (LADs), and dysregulate the active and repressive chromatin domains among different topologically associated chromatin domains (TADs) (Zheng et al., 2018). Lamin mutations also cause increased Yes-associated protein (YAP) nuclear entry in muscle stem cells (Owens et al., 2020) and alter chromatin interactions and depress alternative fate genes (Shah et al., 2021). With the emerging new findings on lamin A/Polycomb Repressive Complex (PRC) crosstalk (Marullo et al., 2016), it is speculated lamin A can also affect gene expression through histone modifications (Bianchi et al., 2018). PRC proteins are known to form PRC aggregates that form an intranuclear structure that regulates long-range chromatin interactions (Lanzuolo et al., 2007; Bantignies et al., 2011; Cesarini et al., 2015). Polycomb Repressive Complex 2 (PRC2) is responsible for H3K27me3 through the catalytic activity of Ezh2 (Bianchi et al., 2018). It is speculated that, under mechanical stress, lamin A is upregulated, leading to more crosstalk with PCR2, resulting in observed accumulation of H3K27me3 (Bianchi et al., 2018). This hypothesis also explains the higher mechanosignaling sensitivity observed in cells carrying lamin A/C mutation: mutated lamin A/C cannot achieve effective crosstalk with PRC2 which further leads to a loss of repressive activity on mechanosensitive genes.

Mechanical stimulation also affects nuclear translocation of transcription factors. Under mechanical stimulation, myocardin-related transcription factors (MRTFs), YAP, and extracellular signal-regulated kinase (ERK) are translocated into the nucleus in myocytes (Wada et al., 2011; Aragona et al., 2013; Velasquez et al., 2013; Gallo et al., 2019). Two mechanisms of NPC mediated nuclear translocation have been proposed: the first hypothesis is that the NPC might increase the ring structure diameter under the mechanical force because of its connection with SUN2 protein. The enlargement of the NPC tunnel promotes the entry of transcriptional factors. The second hypothesis is that the mechanical stimulation changes the state of transcriptional factors which subsequently increase their binding affinities with nuclear transport receptors (Matsuda and Mofrad, 2022). As cells show reduced mechanosensitivity with dysfunction of NPC, both hypotheses could be correct (Mofrad and Kamm, 2009; Fichtman and Harel, 2014).

Emerin, LAP2, and MAN1 proteins are all referred to as LEM proteins. All of them have a LEM domain where they interact with chromatin indirectly *via* BAF (Brachner and Foisner, 2011), which helps facilitate the tethering of chromatin around the nuclear periphery. Patients with EDMD show less repressed chromatin and altered chromatin structure in their skeletal muscle (Ognibene et al., 1999; Fidziańska and Hausmanowa-Petrusewicz, 2003; Mewborn et al., 2010). Under force, emerin monomers are phosphorylated, unbind from BAF, and form oligomers which strengthens their interaction with SUN protein and lamin A/C (Guilluy et al., 2014; Fernandez et al., 2022). Through this mechanism, emerin alters the chromatin organization under force. All of the six LAP2 isoforms can bind with BAF and affect chromatin organization (Wilson and Foisner, 2010). Other than its BAF interactions, LAP2 can also affect chromatin organization through interaction with other nuclear proteins. While other isoforms primarily affect chromatin LAD distribution through interacting with B type lamins, LAP2α only binds with lamin A/C (Dechat et al., 2000b; Naetar et al., 2008). Furthermore, LAP2α also interacts with chromatin-binding behavior of the high-mobility group N protein 5 (HMGN5), and downregulation of LAP2α leads to disorganized HMGN5-targeted chromatin regions (Zhang et al., 2013). In addition to interacting with BAF and lamins, MAN1 can also repress TGFβ, activin, and BMP signaling through its interaction with Smad proteins (Pan et al., 2005). However, this interaction is not myocyte or cardiomyocyte specific. In fact, MAN1’s function in mechanosignaling and in myocytes is understudied. It will be interesting to investigate whether MAN1 can interact with Smad-like proteins in myocytes and to subsequently map out the resulting gene regulatory network.

## 6 Current open questions and remaining challenges

There is a growing body of research that we were not able to include in this review. Nuclear mechanosignaling has been a rapidly developing field of research for decades, and it provides an exciting direction in investigating muscle and heart adaptation, maturation, and disease models. As the majority of the groundwork for understanding the role of nuclear mechanosignaling in cell state and function has been laid in non-muscle cell types, there is now an exciting opportunity to apply this knowledge to investigations into mammalian myocytes. Doing so will reveal novel mechanisms of the mechanical regulation of myocyte structure and function that will be critical in defining disease mechanisms and investigating new therapies for myopathies. Considering the well-established gene editing protocols to edit stem cell-derived myocytes, verifying mechano-sensing protein function and mechanism in human cells is a particularly promising endeavor for future and ongoing research. Moreover, much of our understanding of nucleoskeleton protein function is focused on well-studied proteins like lamins and emerin, while the functions of understudied proteins (such as MAN1) remain far from understood. As such, new research on the variety of other nuclear proteins and their role in regulating nuclear structure, chromatin organization, and nucleoskeletal mechanics will construct a more complete picture of the mechanosignaling processes of healthy and diseased myocytes.

Building upon the rich history of muscle research in which multidisciplinary approaches are embedded, combining multiple existing methodologies and technologies to answer new and exciting questions about myonuclear mechanosignaling will be undoubtedly advantageous. For example, bioinformatics (i.e., quantitative proteomics and transcriptomics) can be applied to future studies to gather a more complete picture of effects of mechanical stimulation and protein mutations in striated muscle mechano-signaling processes. These types of studies will also inform computational investigations into mechanosensitive genetic signaling in myocyte remodeling (Tan et al., 2017; Saucerman et al., 2019; Khalilimeybodi et al., 2023), which will enable a more holistic view of nuclear mechanosensing and myocyte structure/function. Furthermore, studying cells/tissues on a stationary surface/scaffold does not accurately represent the *in vivo* environment of muscles where the cells are constantly experiencing large forces and deformations. Mechanically stimulating the cells to better mimic the innate physical environment can provide more accurate representations of the mechano-sensing protein functions. Exciting ongoing research in these directions is sure to have a great impact on our understanding of the role of the nucleus in mediating muscle function in health and disease.
